# miRNA in situ hybridization in circulating tumor cells - MishCTC

**DOI:** 10.1038/srep09207

**Published:** 2015-03-17

**Authors:** Francisco G. Ortega, Jose A. Lorente, Jose L. Garcia Puche, Maria P. Ruiz, Rosario M. Sanchez-Martin, Diego de Miguel-Pérez, Juan J. Diaz-Mochon, Maria J. Serrano

**Affiliations:** 1 GENYO. Centre for Genomics and Oncological Research: Pfizer/University of Granada/Andalusian Regional Government PTS Granada. Avenida de la Ilustración, 114. 18016 GRANADA, Spain; 2 Laboratory of Genetic Identification, Department of Legal Medicine, University of Granada. Avda. Madrid 11, 18012 Granada, Spain

## Abstract

Circulating tumor cells (CTCs) must be phenotypically and genetically characterized before they can be utilized in clinical applications. Here, we present the first protocol for the detection of miRNAs in CTCs using in situ hybridization (ISH) combined with immunomagnetic selection based on cytokeratin (CK) expression and immunocytochemistry. Locked-Nucleic Acid (LNA) probes associated with an enzyme-labeled fluorescence (ELF) signal amplification approach were used to detect miRNA-21 in CTCs. This protocol was optimized using both epithelial tumor (MDA-MB468) and epithelial non-tumor (MCF-10A) cell lines, and miRNA-21 was selected as the target miRNA because of its known role as an onco-miRNA. Hematopoietic cells do not express miRNA-21; thus, miRNA-21 is an ideal marker for detecting CTCs. Peripheral blood samples were taken from 25 cancer patients and these samples were analyzed using our developed protocol. Of the 25 samples, 11 contained CTCs. For all 11 CTC-positive samples, the isolated CTCs expressed both CK and miRNA-21. Finally, the protocol was applied to monitor miRNA-21 expression in epithelial to mesenchymal transition (EMT)-induced MCF-7 cells, an epithelial tumor cell line. CK expression was lost in these cells, whereas miRNA-21 was still expressed, suggesting that miRNA-21 might be a good marker for detecting CTCs with an EMT phenotype.

Metastasis is responsible for the vast majority of cancer-related deaths^1^. During this process, circulating tumor cells (CTCs) are generated and shed from the primary tumor, colonize distant organs and lead to overt metastatic disease. In the past decade, a growing interest in CTCs has developed among oncology researchers and clinicians because of the potential of CTCs as prognostic elements of cancer^2^,^3^. Despite significant progress in understanding and detecting CTCs, the sensitivity of most assays is low, mainly due to the fact that only a few epithelial biomarkers are used to identify and isolate CTCs from whole blood. EpCAM and cytokeratins (CKs) are the two main epithelial biomarkers that are used in most of the devices that have been utilized to date^4^,^5^,^6^. Among these devices, CellSearch and GILUPI, which have been approved as medical devices by the FDA and the EU, respectively, can detect only EpCAM in circulating cells in the blood^7^,^8^.

However, recent evidence has demonstrated that a subset of CTCs may lack EpCAM and CK expression and instead exhibit features of epithelial to mesenchymal transition (EMT)^9^. Additionally, the use of epithelial biomarkers might lead to the identification of epithelial cells within hematopoietic cell populations that are not derived from tumors but are instead from other epithelial tissues. Accordingly, the development of novel detection platforms should be accompanied by the identification of novel and specific CTC biomarkers that enhance the detection and molecular characterization abilities of these platforms^10^.

MicroRNAs (miRNAs) are small non-coding RNAs that play a key role in the post-transcriptional regulation of mRNA. The relationships between variations in miRNA expression and different pathologies, including different types of cancer^11^, have been described in many reports. miRNAs also circulate within bodily fluids, including peripheral blood and urine, and many studies have reported a correlation between the levels of specific circulating miRNAs and different pathologies, especially cancer^12^. Therefore, miRNAs have been proposed as ideal biomarkers for the development of diagnostic and prognostic liquid biopsy assays. However, the technical difficulties associated with performing robust and comparable profiling of circulating miRNAs across different platforms as well as inter-individual variability, a lack of common internal normalization controls and the unclear functional roles of these miRNAs have impeded the development of an approved clinical diagnostic assay^13^.

To date, there have been many efforts to correlate circulating miRNAs with the number of CTCs^14^. Moreover, in 2011, Sieuwerts profiled miRNAs from the lysates of blood fractions containing CTCs. However, it may be challenging to implement this approach on a broad scale^15^ due to the low number of CTCs in the blood and the issue of leukocyte contamination. Therefore, there is a clear need for an efficient and sensitive method for the detection of miRNA within CTCs.

The aim of this study was to develop protocols to detect CTCs in patient blood samples via miRNA in situ hybridization in CTC (MishCTC) that are combined with simultaneous immunocytochemistry protocols for cell phenotyping. To our knowledge, this is the first report of a protocol that can be used to identify miRNAs in CTCs using in situ hybridization techniques.

## Results

### Integration of LNA-based miRNA-ISH techniques and CTC detection protocols

**Figure 1:**
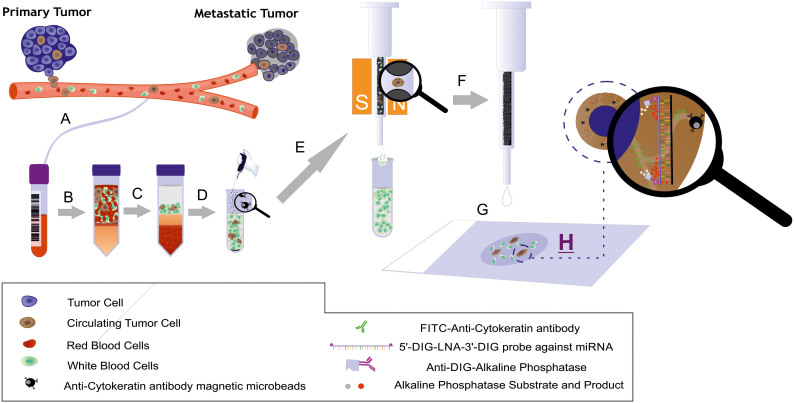
Schematic illustration of the MishCTC method for simultaneous miRNA and CK detection via immunocytochemistry. (A) Recovery of peripheral blood into an EDTA tube; (B) blood transfer into a density-gradient centrifuge tube; (C) centrifugation at 700 × *g* for 30 min; (D) recovery of the interphase layer, which contains mononuclear and tumor cells, and immunomagnetic labeling with magnetic microbeads conjugated to an anti-CK antibody; (E) magnetic cell separation using a MiniMACS separator and a pre-filled separation column; (F) elution of retained cells; (G) application of the cells to a polylysine glass slide using CytoSpin centrifugation; and (H) MishCTC detection of miRNA and CK. Mr. Juan M. Agudo helped to prepare this artwork.

To detect miRNAs in CTCs, we integrated ISH protocols for detecting miRNAs in single cells with the methodological steps necessary to isolate and identify CTCs from patient blood. The initial experiments were performed using a breast epithelial tumor cell line as a model. Briefly, cells were collected from plates and placed on slides via CytoSpin centrifugation. The cells were then treated with EDC^16^ to covalently immobilize the miRNAs in the cytoplasm. Detection was performed via an enzyme-labeled fluorescence (ELF) signal amplification approach^17^ using miRCURY technology, which is based on LNA probes^18^. This technology uses labeled LNA probes, which hybridize to fully complementary miRNA sequences with high affinity. These tags can then be identified via antibodies that are labeled with enzymes that convert fluorogenic enzymatic substrates into fluorescent products. Herein, digoxin (DIG) and a sheep anti-DIG antibody labeled with alkaline phosphatase were used as partners, and FastRed TR was used as a fluorogenic substrate. Upon exposure to alkaline phosphatase enzymatic activity, the FastRed TR substrate produces an insoluble product that can be detected by fluorescence microscopy (Fig. 1).

The breast epithelial tumor cell line MDA-MB468 was used as a model in which to detect miRNA-200, miRNA-21 and U6 in situ by fluorescence microscopy. In addition to the RNAs, nuclei and cytokeratins were also stained with DAPI and FITC-labeled anti-cytokeratin antibodies, respectively (SI Fig. 1 shows the fluorescence images that were obtained using this methodology). The rounded cell morphologies and miRNA distributions are concordant with CytoSpin treatments and with LNA-based ELF detection, respectively^19^. This protocol, which successfully detected RNA via ELF signal amplification in cells that were placed on slides via CytoSpin, was then applied for further experiments.

### Detection of miRNA-21 in blood samples from a healthy volunteer that were spiked with MDA-MB468 cells

**Figure 2:**
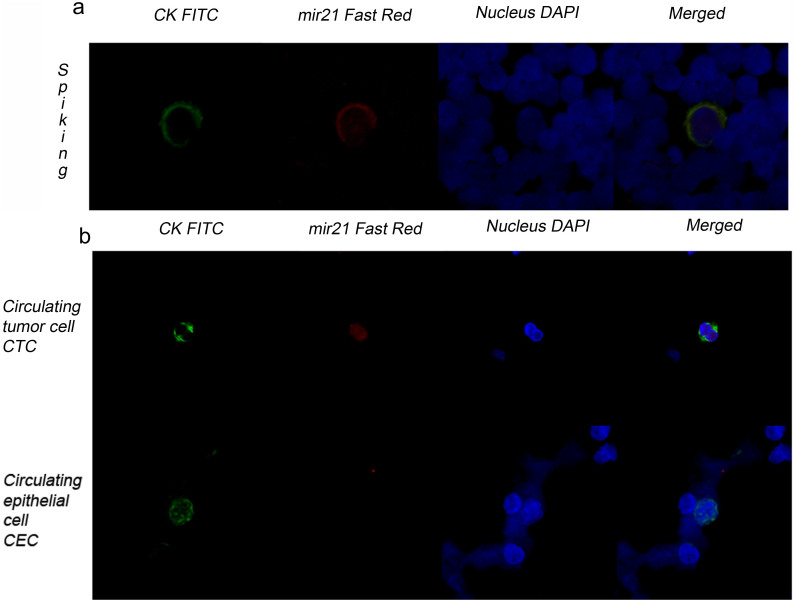
Image galleries obtained with the MishCTC method. (a) CK and miRNA-21 expression in an MDA-MB468 cell that was spiked into a blood sample from a healthy volunteer. Detection of cytokeratin-positive (CK+) cells (green channel), miRNA-21-positive cells (red channel) and nuclei (blue channel). Epithelial cells were identified in a leukocyte population that did not express miRNA-21. (b) CK and miRNA-21 expression in a CTC from a patient with metastatic lung cancer. All the CTCs that were found within this set of patients were both CK- and miRNA-positive (upper panel). CK expression in a circulating epithelial cell from a cancer-free patient undergoing a nephrectomy. CK protein expression (green) was detected by immunofluorescence, but miRNA-21 could not be detected by in situ hybridization (lower panel).

The very low number of CTCs in blood compared to the number of the hematopoietic cells is one of the most challenging aspects for any technology focused on molecular characterization. To optimize the methodological steps that are required to isolate CTCs from blood via positive selection of cells with an epithelial phenotype and subsequently detect miRNAs, we spiked blood samples from 15 healthy volunteers with 100 epithelial cells (MDA-MB468) each. The isolation of cytokeratin-positive cells and their further phenotypic characterization were based on a protocol that was previously established by our group^6^ with minor modifications (see Methods, Fig. 1). After dispensing the cytokeratin-positive cell fractions onto slides via CytoSpin centrifugation, we followed the same protocol described above to identify miRNA-21. We selected miRNA-21 for our in situ experiments because it is one of the most important miRNAs related to cancer development^20^. Moreover, the selected miRNA had an important required feature: it was expressed in tumor cells but not in hematopoietic cells. Thus, CTCs and leukocytes can be easily differentiated using miRNA-21 as a marker. In addition, it is possible that miRNA-21 could be used to differentiate CTCs from normal epithelial cells, as its expression levels in these cells might be different. We isolated an average of 79% of the total spiked cells and found that in every sample, every cell that was cytokeratin-positive also expressed miRNA-21 without exception (SI Table 1). Fig. 2a shows images from a spiking experiment in which a single MDA-MB468 cell was detected amongst the leukocyte population. miRNA-21 can be clearly identified in epithelial cells, whereas it is not detected in leukocytes; thus, it fulfills one of the most important requirements for this assay. These data demonstrate that miRNA-21 expression is a specific biomarker for epithelial cells that does not appear in hematopoietic lineages.

### Quantification of miRNA-21 in MDA-MB468 and MCF-10A cell lines by qRT-PCR

Before proceeding with the analysis of blood samples from cancer patients, we investigated whether miRNA-21 was expressed equally in tumor and non-tumor epithelial cells. We therefore performed both in situ hybridization of miRNA-21 according to previously established protocols and a miRNA-21 expression analysis by qRT-PCR in a breast cancer cell line (MDA-MB468) and a normal breast cell line (MCF-10A). The fluorescence intensities per cell and ddCT values from both of the cell lines confirmed that miRNA-21 is overexpressed in epithelial tumor cells compared with non-tumor cells (see Methods, SI Fig. 2 and SI Fig. 3). These results confirm that miRNA-21 is a valid biomarker that can be used to differentiate CTCs from leukocytes and tumor from non-tumor epithelial cells.

### Detection of miRNA-21 in CTCs from blood samples of cancer patients

To evaluate the potential implementation of this method as a diagnostic tool in patient blood samples, we used peripheral blood samples obtained from 25 patients with metastatic cancer who provided informed consent (SI Table 2). All the samples, including those from healthy donors, were treated and analyzed as described above. CTCs were detected in 11 patients, and in every case, CTCs were identified concomitantly by CK and miRNA-21 expression (Fig. 2b and SI Table 2). All the samples from healthy donors were negative for both CK and miRNA-21 expression. Finally, to confirm that miRNA-21 is expressed differently in circulating tumor and non-tumor epithelial cells, a blood sample from a cancer-free patient who recently underwent a nephrectomy was used as a source of circulating non-tumor epithelial cells. Fig. 2b shows microscopy images of that sample following our MishCTC protocol. In this case, the epithelial cells did not show miRNA-21 expression, although they maintained an epithelial cytokeratin phenotype.

### Detection of miRNA-21 in EMT-induced MCF-7 cells

We used the MishCTC protocol to investigate miRNA-21 expression in the EMT-induced MCF-7 cell line, an epithelial tumor cell line, as a model of the heterogeneity of CTCs that was recently reported by our group^21^ Kubo et al.^22^ and Paramio et al.^23^ reported that miRNA-21 is induced in epithelial cells undergoing EMT, and standard markers, such as cytokeratin, are lost in these cells. We therefore investigated whether our method could be used to detect heterogeneous epithelial cell lines that had lost epithelial biomarkers, such as cytokeratin, but still maintained expression of miRNA-21 as reported by Kubo et al.^22^ and Paramio et al.^23^. MCF7 cells were plated in 96-well plates and induced with TGF-β as previously reported by our group^21^. SI Fig. 4 presents miRNA-21 and CK expression in both MCF-7 and TGF-β-induced MCF-7 cell lines that were labeled using the MishCTC protocol. Unmodified MCF-7 cell lines expressed miRNA-21 in a heterogeneous manner within the same cell culture, and this heterogeneity was not observed previously in MDA-MBA468 cells. In the TGF-β-induced MCF-7 cell line, as expected, there was a population of cells that had lost CK expression but maintained miRNA-21 expression, generating cells that were CK-negative and miRNA-21-positive.

## Discussion

We present the first protocol for detecting miRNAs in CTCs using an ISH technique called MishCTC. This protocol is compatible with immunocytochemical detection of protein markers and uses LNA-based probes with an ELF signal amplification approach. The MishCTC protocol was generated via the integration of ISH techniques with standard protocols for CTC enrichment from blood samples based on immunomagnetic positive selection of epithelial cells. Using this protocol, the expression of miRNA-21, an onco-miRNA, was determined. A total of 25 patients were recruited, 11 of which presented CTCs with CK expression. The expression of miRNA-21 was restricted to epithelial tumor cells in the peripheral blood of these patients and was absent from the leukocytes. One of the challenges of working with CTCs is that contamination with hematopoietic cells is always present, even after enrichment. However, miRNA-21 is not expressed in leukocytes, which is a major advantage. This protocol was also used to analyze EMT-induced cell lines and was capable of identifying cells that were negative for epithelial markers (CK-) and positive for miRNA-21 expression. Currently, we are recruiting a larger cohort of cancer patients to investigate CTC heterogeneity based on miRNA expression using the MishCTC protocol.

The detection and characterization of CTCs is complex, and many groups studying the molecular and phenotypic characteristics of CTCs are aiming to better understand metastatic processes. Some researchers have examined the correlation between the expression of miRNA (as detected in the tissue or serum) with the presence or absence of CTCs. However, the detection of miRNA in CTCs via in situ methods has not been previously reported. These protocols can also be used to better understand the molecular mechanisms that are associated with dissemination and metastatic processes.

This report is the first proof of concept for MishCTC, which will be used to analyze multiple miRNAs in CTCs. MishCTC protocol is thus a potential tool that can be used to monitor cancer patients and determine the efficacy of their treatments.

## Methods

All the experiments were performed in accordance with the relevant guidelines and regulations.

### Cell culture

Breast cancer cell lines were obtained from the American Type Culture Collection (ATCC, Manassas, VA, USA). The MDA-MB468 tumor cells were maintained in DMEM culture medium (Gibco, Paisley, UK) that was supplemented with 10% fetal bovine serum (Gibco), 100 U ml^−1^ penicillin and 100 ng ml^−1^ streptomycin at 37°C in a humidified incubator with 5% CO_2_. MCF-10A non-tumor cells were maintained in serum-free mammalian epithelial growth medium (MEGM) (Clonetics® Lonza, South Plainfield, NJ, USA) with 100 ng mL^−1^ cholera toxin (Sigma-Aldrich, Saint Louise, MO, USA).

### Preparation of breast cancer cell lines via CytoSpin for the simultaneous detection of cytokeratins and miRNAs

One million cells were seeded in a 75-cm^2^ treated flask (NUNC™, Roskilde, Denmark) in their corresponding cell culture media. After incubation for 72 h, each well was washed with PBS, and the cells were trypsinized with 0.05% trypsin in 1× PBS for 15 min, neutralized with soybean trypsin inhibitor (0.1% trypsin inhibitor in 1× PBS) and resuspended in PBS (pH 7.4). The cell suspensions were then permeabilized with 10% MACS Cell Perm Solution (Miltenyi Biotec, Bergisch Gladbach, Germany) for 5 min and fixed with 10% MACS Cell Fix Solution (Miltenyi Biotec)or 30 min followed by washing three times with 1× PBS (5 min each wash). The cell pellets were resuspended in 1× PBS and spun down onto polylysine-coated glass slides (Sigma-Aldrich, Gillingham, UK) using a cytocentrifuge (Hettich, Germany) at 1,500 rpm for 10 min. The slides were air-dried overnight at room temperature and stored at 4°C. The dried slides containing cells that were immobilized by CytoSpin were rehydrated with 1× TBS buffer for 5 min and treated twice (5 min each treatment) with 100 μl of a 130 mM aqueous solution of 1-methylimidazole (AppliChem, Darmstadt, Germany). The areas of interest were then marked with a hydrophobic pen (Dako, Glostrup, Denmark). Subsequently, 100 μl of a 160 mM aqueous solution of 1-ethyl-3-(3-dimethylaminopropyl)carbodiimide (EDC) (Sigma-Aldrich, UK) was added, and the slides were placed in a humidity chamber (ThermoBrite system, Abbot Molecular, Des Plaines, IL, USA) for 1 h. Then, the slides were washed twice for 5 min with 1× PBS before incubation in a hybridization chamber for 30 min with 100 μl of Proteinase QS solution (1:8000 dilution in 1× PBS) (Affymetrix, Santa Clara, CA, USA). The slides were then washed twice for 5 min with 1× PBS before being dehydrated with increasing ethanol concentrations (70%, 96% and 99.5%; 1 min at each concentration) (Sigma–Aldrich, UK).

### LNA-based detection probes

Locked nucleic acid (LNA)-based oligonucleotides for the detection of miRNA-21, miRNA-200a and U6 snRNA were purchased from Exiqon (Vedbæk, Denmark) as miRCURY LNA™ miRNA kits. These oligonucleotides are 20-mer oligonucleotides that are labeled at both the 5′ and 3′ ends with digoxin (DIG).

### Simultaneous detection of cytokeratins and miRNAs by fluorescent immunocytochemistry and ISH techniques (MishCTC)

The expression of miRNA-21, miRNA-200a and U6 snRNA was determined using miRCURY LNA™ miRNA kits (see above). Each probe was independently analyzed. After dehydration, the slides were air-dried and incubated with 40 μl of a diluted solution (1:600) of the corresponding LNA miRNA probe, which was pre-denatured by heating at 90°C for 4 min in 1 × ISH buffer (Exiqon) and hybridized at 58°C in a humidified chamber for 1 h after sealing the samples with fixogum. Following hybridization and the removal of the fixogum, the slides were washed with 5×, 1× and 0.2× SSC (5 min each wash) at 56°C. The samples were then incubated for 15 min in a blocking solution (0.1% Tween, 2% sheep serum and 1% BSA in 1× PBS) followed by incubation for 15 min in a solution containing both a FITC-anti-cytokeratin antibody (clone: CK3-6H5; Miltenyi Biotec) and an anti-DIG alkaline phosphatase antibody (Roche Diagnostics, Germany). After incubation with both of the antibodies, the samples were washed with 1× PBS for 5 min. Enzyme-labeled fluorescence (ELF) signal amplification was achieved by applying SIGMAFAST™ FastRed TR/Naphthol (Sigma-Aldrich, UK), diluted in Tris-HCl buffer according to the manufacturer's recommendations, as a substrate for alkaline phosphatase. Finally, VECTASHIELD mounting medium with DAPI (Vector Labs, Burlingame, CA, USA) was used to mount the slides.

### Spiking experiments

Spiking experiments were performed in triplicate by dispensing 100 MDA-MB 468 cells into 10-ml venous blood samples that were collected from 20 healthy volunteers in 10-ml EDTA tubes (BD, Franklin Lakes, NJ, USA). The samples were processed by density gradient centrifugation for 45 min at 400 rpm. Histopaque®-1119 (Sigma-Aldrich, UK) was used to isolate the hematopoietic cell fractions, which also contain epithelial cells. The hematopoietic fractions were then incubated for 30 min with magnetic microbeads that were labeled with a multi-cytokeratin-specific antibody (CK3-11D5) (Miltenyi Biotec) that recognizes cytoplasmic cytokeratins 7, 8, 18 and 19. The magnetically enriched cell fractions were then passed through MACS Cell Separation magnetic columns (Miltenyi Biotec) that were supported by a MiniMACS separator (Miltenyi Biotec) and washed three times with dilution buffer (Miltenyi Biotec). The magnetic columns were then detached from the MiniMACS separator support, and the cytokeratin-positive cells were eluted from the column after adding dilution buffer and applying pressure. The cytokeratin-positive-enriched cell fractions were spun down onto polylysine-coated glass slides. Then, previously described protocols were utilized for the simultaneous detection of cytokeratins and miRNAs. The recovery rates of tumor cells spiked into normal blood at low levels ranged from 60% to 75%.

### Analysis of CTCs from patient samples

A total of 25 patients with metastatic cancer from the Breast Cancer Unit of the University Hospital of Granada were enrolled in the study from December 2013 to January 2014. The inclusion criteria were the histological diagnosis of lung cancer (LC), prostate cancer (PC), colon cancer (CC), urothelial cancer (UC), breast cancer (BC), gastric cancer (GC), ovarian cancer (OC) or melanoma (MC) and the availability of tissue for biomarker studies (SI Table 2). This translational study was approved by the Independent Ethics Committee of San Cecilio Hospital (Granada, Spain). Following the approval of the study by The Ethics Committee, eligible patients were selected and informed written consents were provided.

As negative controls, 5 blood samples from healthy volunteers without evidence of epithelial malignancy were examined. Peripheral blood was drawn from the middle of the vein puncture, and the first 3 ml of blood was discarded to avoid contamination of the sample with non-tumor epithelial cells from the skin. Blood samples were collected in EDTA tubes and transported immediately to the laboratory. Then, the protocols described in the “*Spiking experiments*” section were performed.

### RNA extraction, reverse-transcription and qRT-PCR

The expression of miRNA-21 and miRNA-200a was analyzed in breast cancer cell lines. The cells (1×10^3^) were plated in a 75-cm^2^ treated flask (NUNC™, Roskilde, Denmark) and grown in culture medium for 72 h. Subsequently, the cells were treated for 3 min with trypsin solution (Sigma Aldrich, US), and 5 ml of medium was added to inhibit the tryptic activity, which may damage the cells. Next, the cells were centrifuged at 200 × *g* for 5 min, collected in 15-ml tubes, and washed once with 1× PBS prior to the addition of 0.5 ml of miRNA Extraction Kit Lysis Mixture (Omega Bio-Tek, Norcross, GA, USA) per tube. The tubes were then incubated for 5 min at room temperature. Then, 250 μl of XD binding buffer was added, and the tubes were incubated on ice for 10 min. After this incubation period, the solution was added to a Hi-Bind® X-press column and centrifuged for 1 min at 13,000 × *g*. Then, 1.2 volumes of ethanol were added to the filtrate, which was transferred to a Hi-Bind® MicroRNA column and centrifuged again at 13,000 × *g* for 1 min. The filtrate was then discarded, and the miRNAs were retrieved from the columns via the addition of 500 μl of RNA wash buffer. The miRNA that was retrieved from the columns was mixed with 5 μl of extracted miRNA and incubated with 1 μl of poly(A), 2 μl of tailing buffer and 2 μl of nuclease-free water (Quanta BioSciences, Gaithersburg, MD, USA). This mixture was incubated at 37°C for 60 min, followed by a 5-min incubation at 70°C. For RT-PCR, a reaction mix was prepared by mixing 25 μl of PerfeCTa SYBR Green Supermix (ROX) with primers (200 nM) and template in a final volume of 50 μl. RT-PCR was performed in triplicate in 96-well plates using a Real-Time PCR System (Applied Biosystems® 7500 Real-Time PCR System, Drive Foster City, CA, USA). After an initial denaturation at 95°C for 2 min, 40 cycles of denaturation at 95°C, annealing at 60°C for 1 min, and extension at 70°C for 1 min were carried out.

### TGF-β−induced EMT cell lines

These methods were previously reported by our group^21^.

### Confocal microscopy

Confocal images were obtained using a Zeiss LSM 710 confocal/multiphoton laser scanning microscope that was equipped with an Argon/2 laser (458 nm, 477 nm, 488 nm, and 514 nm) and a Titanium Sapphire laser (750 nm). The cells were viewed with a ×63 (NA1.2) apochromatic water objective, and images of different fields were obtained. The microscope was set up to obtain multichannel images, and the excitation and emission filter sets were configured individually to ensure no fluorescence bleed-through between the channels. An argon laser was used to visualize FITC with excitation at 488 nm and FastRed with excitation at 543 nm. The appropriate emission filters were used for each fluorophore. FITC was used to label CK, and FastRed/Naphthol was used to label each analyzed miRNA. Zen 2009 light edition software (Carl Zeiss MicroImaging GmbH) was used for image processing and for control of the microscope, the scanning module, and the laser module.

## Author Contributions

F.G.O. performed and optimized the protocols, handled the blood samples, performed microscopy and q.R.T.-P.C.R. assays and analyzed the data. M.P.R. performed the technical work. DMP performed E.M.T. assays. J.L.G.P. monitored the experiments and provided the patient samples. M.J.S. and J.J.D.M. wrote the paper. M.J.S., J.A.L., R.M.S.M. and J.J.D.M. designed and directed the work.

## Supplementary Material

Supplementary Information miRNA in-situ hybridization in Circulating Tumor Cells - MishCTC
