# Supplementary material for: miRNA in situ hybridization in circulating tumor cells - MishCTC
Source: Sci Rep. 2015 Mar 17;5:9207. doi: 10.1038/srep09207 (PMC4361864; doi:10.1038/srep09207)
Supplement: Supplementary Information — miRNA in-situ hybridization in Circulating Tumor Cells - MishCTC [file srep09207-s1.pdf]

Supplementary Information

miRNA in-situ hybridization in Circulating Tumor Cells - MishCTC

Francisco G. Ortega[1], Jose A. Lorente[1,2], Jose L. Garcia Puche[1], Maria P. Ruiz[1], Rosario M. Sanchez-Martin [1], Diego de Miguel-Pérez[a1], Juan J. Díaz-Mochon[1]* and Maria J. Serrano[1]

1GENYO. Centre for Genomics and Oncological Research: Pfizer/University of Granada/Andalusian Regional Government PTS. Granada. Avenida de la Ilustración, 114. 18016 GRANADA, Spain,

2. Laboratory of Genetic Identification, Department of Legal Medicine, University of Granada. Avda. Madrid 11, 18012 Granada, Spain

* To whom the correspondence should be addressed. Corresponding author: mjose.serrano@genyo .es, juanjose.diaz@genyo.es

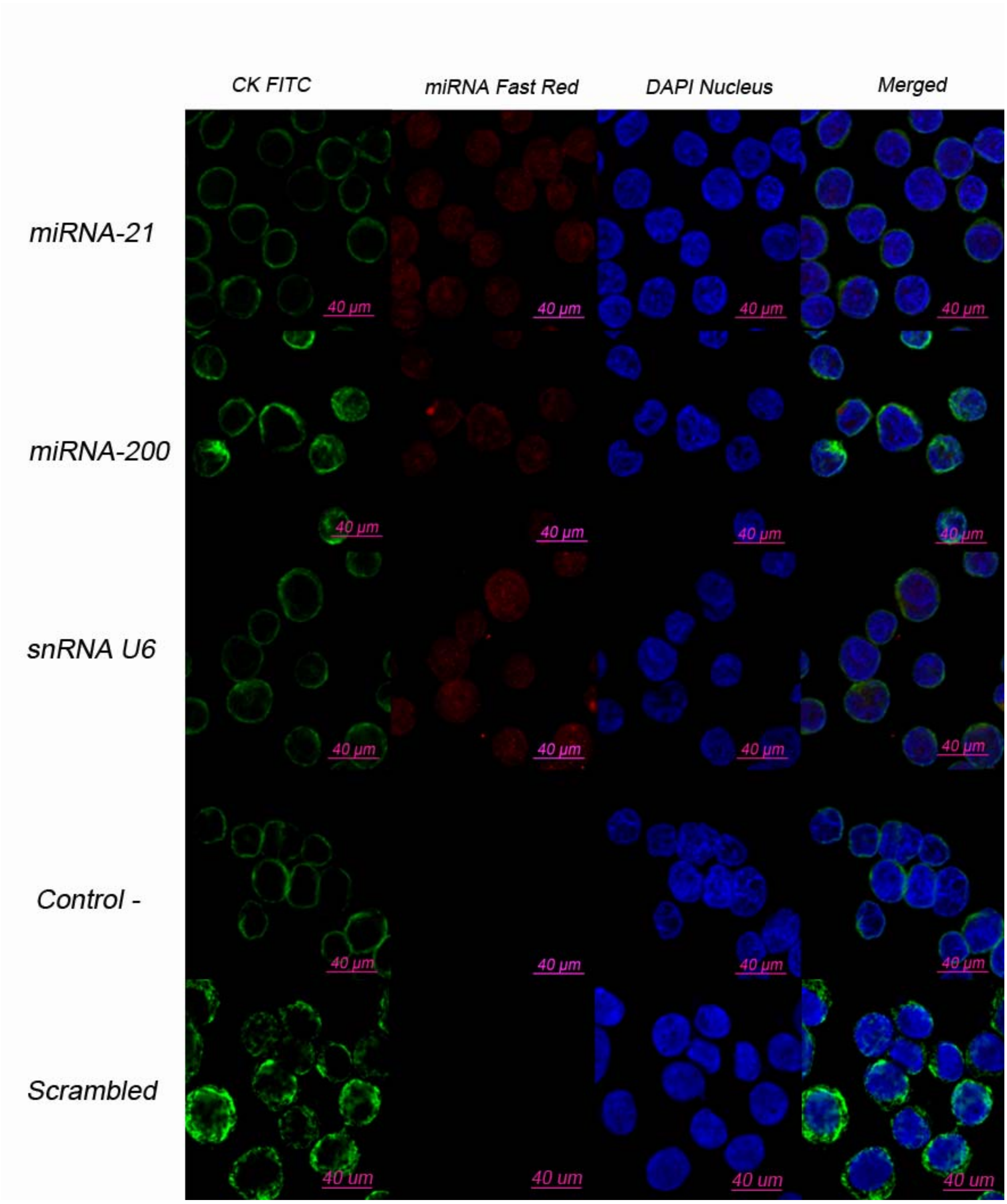

SI Figure 1. Summary of fluorescent images showing miRNA sequences and cytokeratins expressed in MDA-MB468 tumor cell line using locked nucleic acid (LNA) probes labeled with digoxigenin and anti-CK antibody FITC. miRNA and cytokeratin were thus detected by both in situ hybridization of miRNA and immunofluorescence technique. Rows show cytokeratin (green channel), miRNAs (red channel), nuclei (blue channel - Dapi) and merged images. Each row corresponds to detection of miRNA-21, miRNA-200, snRNA U6 and control (none LNA

probe was added) from top to bottom. LNA™ scrambled microRNA probe, double-DIG labeled . LNA™ (5’-gtgtaacacgtctatacgccca-3’) was used as negative control.

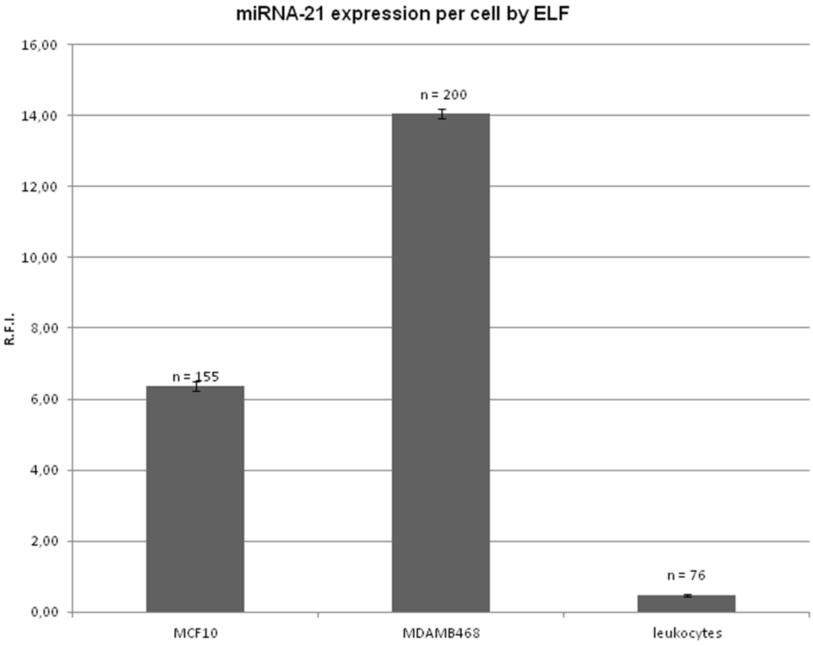

SI Figure 2. Mean fluorescence intensities of miRNA21 in MDA-MB468, MCF10A, and leucocytes generated by ELF signal amplification using LNA probes. Quantification was performed using Image J software. miRNA-21 was over-expressed in MDA-MB468 cell tumor line if compared with epithelial non tumor cell line MCF10A. None fluorescence signal was observed in leucocytes.

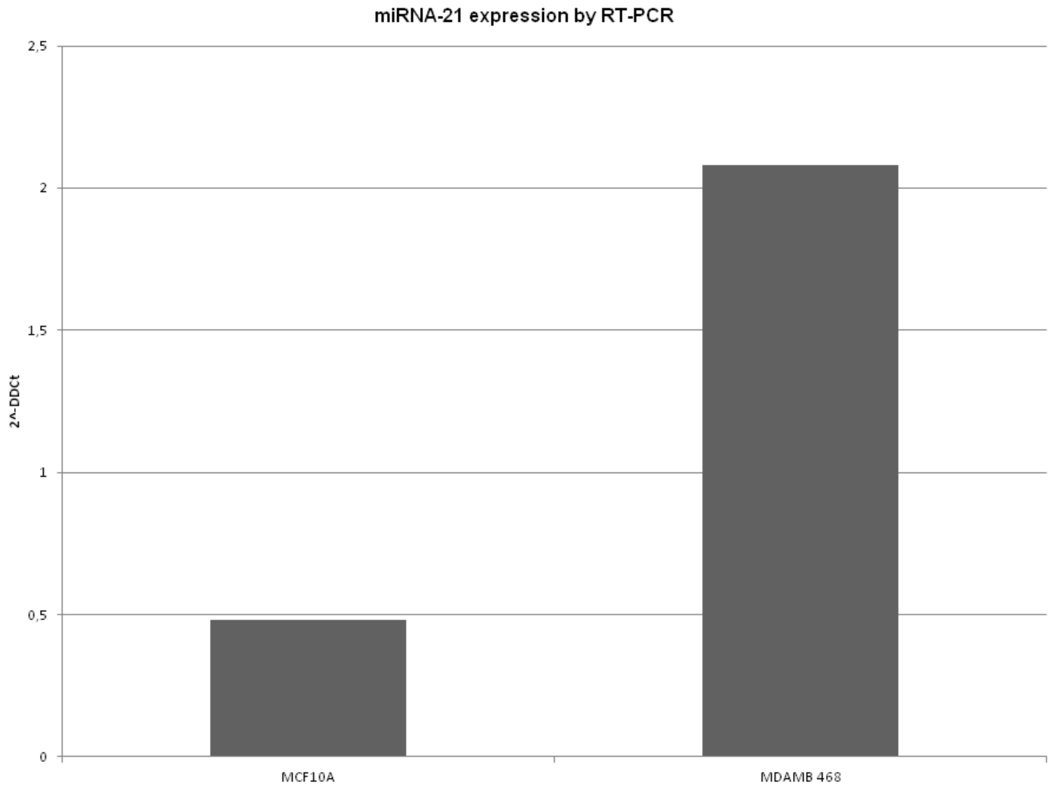

SI Figure 3. Expression of miRNA-21 by RT-PCR. These experiments showed a relative higher expression of miRNA21 in MDA-MB468 than in MCF10A. The molecular analysis by RT-PCR corroborates the potential value of miRNA21 to differentiate circulating epithelial tumor cells from epithelial non-tumor cells.

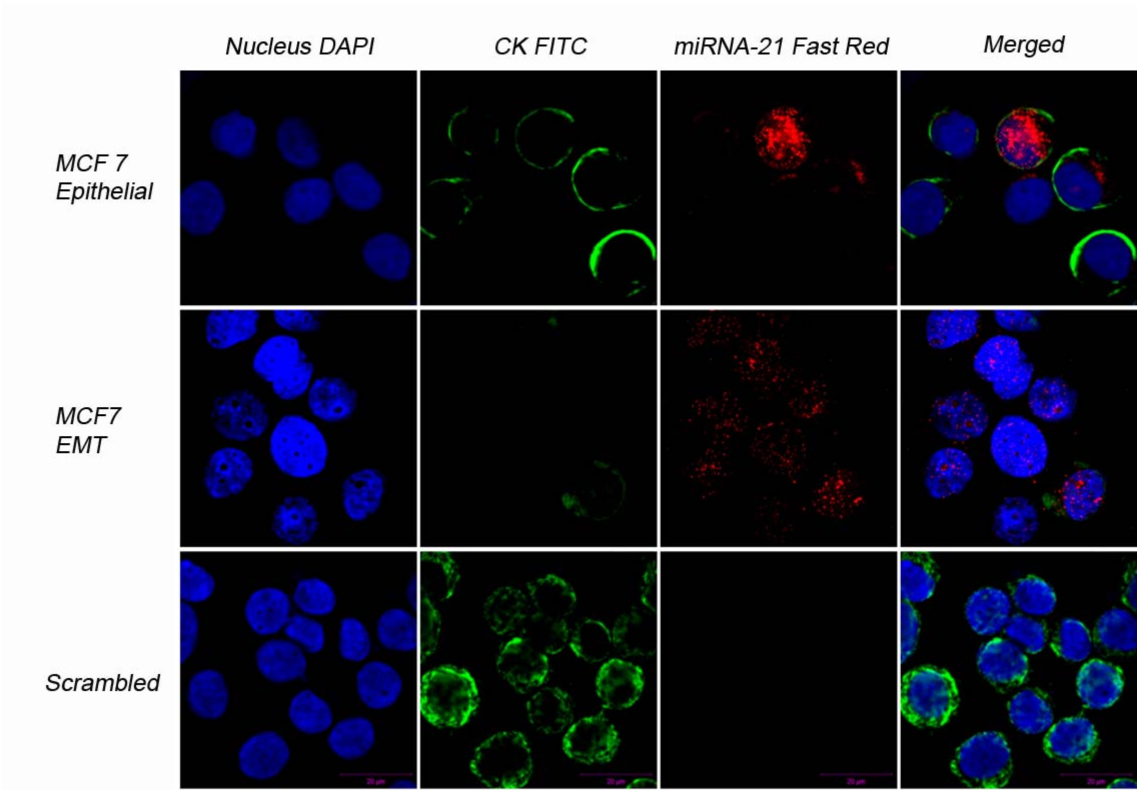

SI Figure 4. Summary of fluorescent images showing miRNA sequences and cytokeratins expressed in MCF7 tumor cell line using locked nucleic acid (LNA) probes labeled with digoxigenin and anti-CK antibody FITC. miRNA and cytokeratin were thus detected by both in situ hybridization of miRNA and immunofluorescence technique. Upper panel show cytokeratin (green channel), miRNAs (red channel), nuclei (blue channel - Dapi) and merged images in MCF7 cell tumor line. Middle panel show cytokeratin (green channel), miRNAs (red channel), nuclei (blue channel - Dapi) and merged images in MCF7 under EMT process. A scramble sequence was used as negative control (lower panel)

SI Table 1. Number of MDA-MB468 cells recovered from spiking 10 ml of healthy volunteer blood sample with 100 MDA-MB468 cells .

**Sample**	**Number of identified cells***
1	74
2	82
3	67
4	90
5	72
6	87
7	81
8	71
9	62
10	64
11	76
12	70
13	84
14	62
15	66
**Average**	*73.86*
**Std Error**	*1.28*

SI Table 2. Anatomic pathological characteristics of cancer patients and number of circulating epithelial cells with expressing CK and miRNA-21.

Patient Code	TUMOR TYPE	METASTATIC TISSUE	HISTOLOGY	CK+ miRNA-21 -	CK+miRNA-21+
1	COLON	LIVER	ADENOCARCINOMA	0	3
2	BREAST	BONE	ADENOCARCINOMA	0	0
3	MELANOME	SKIN		0	0
4	LUNG	BONE	SQUAMOUS CELL CARCINOMA	0	0
5	COLON	TORAX AND LIVER	ADENOCARCINOMA	0	0
6	LARINGE	LYMPH NODES	SQUAMOUS CELL CARCINOMA	0	1
7	BREAST	THORACIC WALL	CDI GII	0	0
8	COLON	LUNG	ADENOCARCINOMA GII	0	0
9	OVARY	ABDOMEN	SEROUS CARCINOMA	0	0
10	COLON	ILIAC CREST	MUCOSECRETOR ADENOCARCINOMA	0	0
11	COLON	OVARY AND UTERUS	ADENOCARCINOMA GII	0	2
12	COLON	LIVER	ADENOCARCINOMA	0	1
13	LUNG	PLEURAL CAVITY	ADENOCARCINOMA	0	0
14	OVARY	ABDOMEN	INDIFFERENT CARCINOMA	0	1
15	PROSTATE	BONE AND MEDIASTINUM	ADENOCARCINOMA	0	7
16	GASTRIC	LIVER	ADENOCARCINOMA GII	0	0
17	BREAST	BONE	CLI	0	0
18	COLON	LIVER AND LUNG	ADENOCARCINOMA GIII	0	0
19	LUNG	MEDIASTINUM	SQUAMOUS CELL	0	6
20	OVARY	LUNG AND LYMPH NODES	MIXED EPITHELIAL ADENOCARCINOMA	0	2
21	COLON	LIVER	ADENOCARCINOMA	0	2
22	LUNG	LYMPH NODES	SMALL CELL LUNG CARCINOMA	0	0
23	COLON	LUNG AND LIVER	MUCOSECRETOR ADENOCARCINOMA	0	1
24	UROTHELIAL	ABDOMEN AND LYMPH NODES	PAPILLARY UROTHELIAL CARCINOMA	0	1
25	ADRENAL	LIVER AND LUNG	ADRENOCORTICAL CARCINOMA	0	0
